# Social Contact with Family and Non-Family Members Differentially Affects Physical Activity: A Parallel Latent Growth Curve Modeling Approach

**DOI:** 10.3390/ijerph18052313

**Published:** 2021-02-26

**Authors:** Yuta Nemoto, Ryota Sakurai, Hiroko Matsunaga, Yoh Murayama, Masami Hasebe, Mariko Nishi, Miki Narita, Yoshinori Fujiwara

**Affiliations:** Research Team for Social Participation and Community Health, Tokyo Metropolitan Institute of Gerontology, Tokyo 173-0015, Japan; r_sakurai@hotmail.co.jp (R.S.); hirokomatsunaga719@gmail.com (H.M.); yhoyho05@tmig.or.jp (Y.M.); hasebe@tmig.or.jp (M.H.); nishi@tmig.or.jp (M.N.); mwata@tmig.or.jp (M.N.); fujiwayo@tmig.or.jp (Y.F.)

**Keywords:** physical activity, physically active, social support, social contact, exercise, parallel latent growth curve modeling, longitudinal study

## Abstract

Background: Social contact leads to an increased likelihood of engaging in physical activity (PA). However, the influence of social contact on PA would be different depending on the social contact source. This study aimed to identify the association of changes in social contact with family and non-family members with the change in PA using a parallel latent growth curve modeling. Methods: Participants were randomly selected from among residents in the study area age ≥ 20 years (*n* = 7000). We conducted mail surveys in 2014, 2016, and 2019. The 1365 participants completed all surveys. PA was assessed with validated single-item physical activity measure. Social contact was assessed by summing frequencies of face-to-face and non-face-to-face contacts with family/relatives not living with the participant and friends/neighbors. Parallel latent growth curve modeling was used to assess the cross-sectional, prospective, and parallel associations of social contact with PA change. Results: There was a positive cross-sectional association between contact with friends/neighbors and PA, whereas prospective and parallel associations between contact with family/relatives and PA. Conclusion: Contacting friends/neighbors did not predict the change in PA, and a high frequency of contact with family/relatives at baseline and increasing contact with family/relatives was associated with increased PA over 5-year.

## 1. Introduction

The health benefits of physical activity (PA) and exercise are well established. Lifelong PA plays a critical role in preventing chronic disease and maintaining functional capacities [1,2,3,4].

The proportion of Japanese adults who exercise regularly is considerably low and decreasing [5]. According to a Japanese national survey, the age-adjusted rate of individuals who performed exercise for more than 30 min at least twice a week over the previous year decreased slightly from 29.8% (men) and 24.5% (women) in 2008 to 27.2% (men) and 21.1% (women) in 2018. This rate is especially low among young adults [6]. These data indicate the need to detect the determinant factors of PA and develop strategies for increasing population-level PA by strengthening access to and promoting participation in sports and active recreation across all age groups [2].

Existing work has reported that multi-level factors affect PA level [7]. Social factors such as social support and social networks play a critical role in promoting PA [8,9,10]. Although social contact contributes to expanding the social network, a social support source, a few studies have investigated the association of social contact with PA [11,12]. Social contact would have both direct and indirect effects on PA. As a direct effect, individuals with a high frequency of social contact may experience a greater sense of connectedness, which may motivate individuals to take better care of their health and lead them to exercise together with their peers [13,14]. As an indirect effect, individuals with a greater degree of social contact may have improved mental health, which is one of the determinants of PA [15,16]. Since most studies focusing on these points have been cross-sectional, [17], a prospective cohort study with a population-representative sample is needed to identify longitudinal associations of social contact with PA. Furthermore, previous cross-sectional studies have reported the associations of social contact with PA are differentially between the social contact sources, concluding that contacting friends is associated with a higher PA level than family contact [18]. The longitudinal association of these behaviors remains unclear, and identifying the social contact resource that increases PA would contribute to developing the effective PA promoting intervention.

Additionally, recent studies have examined the prospective and parallel associations of exposure with the outcome [19,20,21]. This approach allows estimating the relationship between changes in exposure and changes in outcome. Given that social contact and PA change over the life course [22,23], examining the association of the social contact trajectory with PA can help understand the dynamic relationships between these behaviors.

Hence, in this study, we aimed to investigate the longitudinal association of social contact with PA, distinguishing social contact sources (family/relatives, friends/neighbors) among community-dwelling adults. We conducted a longitudinal study, collecting data at three time points, to examine the 5-year trajectories of social contact and PA and the prospective and parallel associations.

## 2. Materials and Methods

### 2.1. Study Design and Participants

A survey with a longer follow-up period and assessment at multiple time points was desirable to examine the longitudinal and parallel relationships between social contact and PA. Therefore, we conducted a 5-year prospective cohort study enrolling residents of an urban area in Japan.

The baseline and follow-up surveys were conducted in October 2014 (wave 1), November 2016 (wave 2), and October 2019 (wave 3). Surveys were performed in Wako city, Saitama, Japan, which had a population of 79,338 and a population aging rate of 16.2% in 2014.

In wave 1 (2014), eligible survey participants were randomly selected, stratified according to age group, from among residents age ≥ 20 years. A total of 7000 adults were invited to participate in the baseline survey. Each age group’s sample size was as follows: young adults aged <40 years, *n* = 3000; middle-aged adults aged 40–64 years, *n* = 3000; older adults aged ≥ 65 years, *n* = 1000. We excluded residents receiving long-term care insurance services at levels 4 and 5, requiring assistance with daily living. In follow-up surveys (waves 2 and 3), enrolled the previous survey respondents, excluding individuals who died, moved from the study area, or received long-term care insurance services at levels 4 and 5 before the survey.

**Figure 1:**
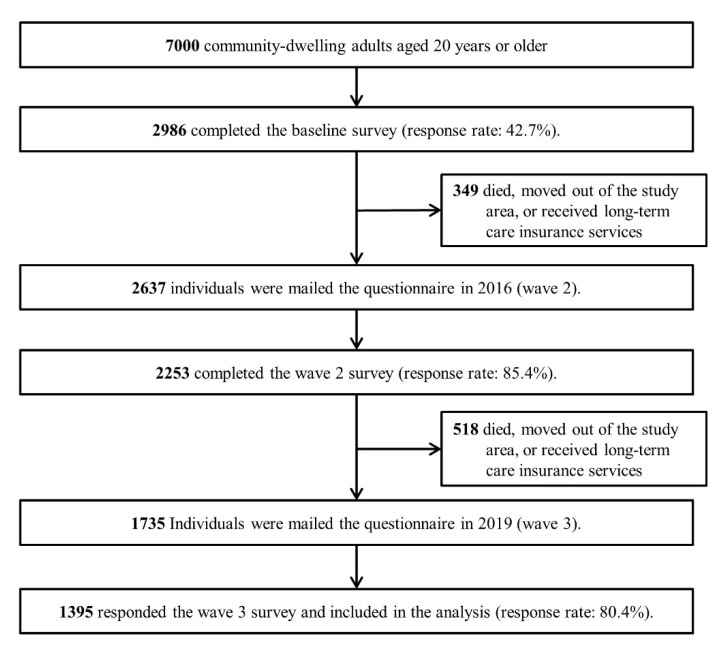
Flow diagram of this study.

In each wave, participants were asked to complete a self-administered questionnaire. The number of respondents was as follows: wave 1, *n* = 2986; wave 2, *n* = 2253; and wave 3, *n* = 1395. The participants who completed all surveys were included in the analysis (*n* = 1395) (Figure 1).

Informed consent was obtained from all participants, with ethical approval granted by the Ethics Committee of the Tokyo Metropolitan Institute of Gerontology (protocol code: 1183, date of approval: 17 July 2019).

### 2.2. Measurements

#### 2.2.1. Physical Activity

Although the PA guidelines provide a recommendation concerning the amount (frequency, intensity, duration) of PA [24], this study evaluated PA with only the frequency. In each wave, PA was assessed with a single-item physical activity measure, representing strong repeatability and moderate validity [25]. Participants were asked about the frequency of physical activity, which was enough to raise the breathing rate, for a total of 30 min or more. The frequency was reported from 1 (almost every day) to 8 (never). As the average weeks in a month is 4.3 weeks, the amount of PA per month was computed as follows: 6–7 times/week = 28.0 times; 4–5 times/week = 19.4 times; 2–3 times/week = 10.8 times; 1 time/week = 4.3 times; 2–3 times/month = 2.5 times; 1 time/month = 1 time; infrequently or never = 0.

#### 2.2.2. Social Contact

The frequencies of face-to-face and non-face-to-face communication were evaluated for family members and relatives not living with participants (family/relatives) and with friends or neighbors (friends/neighbors) [26]. For evaluation of in-person contacts, participants were asked to report the frequency of encountering or spending time with family or non-family members; non-in-person contact was evaluated using participants’ reported frequencies of telephone contact or emailing with family or friends/neighbors. The frequencies were reported from 1 (almost every day) to 8 (never) for each item; the frequencies of face-to-face and non-face-to-face contacts were then summed for the total, for family/relatives, and for friends/neighbors. The number of social contacts per month (4.3 weeks) was calculated as follows: 6–7 times/week = 28.0 times; 4–5 times/week = 19.4 times; 2–3 times/week = 10.8 times; 1 time/week = 4.3 times; 2–3 times/month = 2.5 times; 1 time/month = 1 time; less than once a month = 0.5 times; never = 0.

### 2.3. Covariates

Based on previous studies, variables that would be a common cause of social contact and PA, such as baseline sociodemographic variables [18,20,27], health status [27,28], and health behaviors [29], were included as covariates.

Sociodemographic variables included sex, age, years of education (<13 years or ≥13 years), subjective economic status, marital status (married, widowed/divorced/single), and employment status. Economic status was self-reported and classified as high (those reporting financial security), low (those reporting financial insecurity), and medium (neither) categories. Although household income was also investigated, the proportion of missing information was high (15.9%). The distribution of household income varies depending on the age group [30]; for these reasons, we included subjective economic status as a covariate. Employment status was assessed based on the type of occupation: non-workers, students, and homemakers were classified as not in the labor force; other occupations were classified as a paid worker.

Health conditions included self-rated health status (good or poor), mental health status, and medical history of diabetes mellitus and hypertension (yes or no). As a measure of mental health, we used the simplified Japanese version of the World Health Organization-Five Well-Being Index (S-WHO-5-J) [31]. The measure was simplified from the WHO-Five Well-Being Index. The total score ranged from 0 to 15, with higher scores indicating a better mental health condition.

Health behaviors included alcohol consumption (≥1 time/week, <1 time/week, former drinker, non-drinker), smoking status (current smoker, former smoker, non-smoker), and eating habits. Eating habits were rated according to the reported frequency of having a balanced diet twice per day in an average week; this was categorized as ≥5 days/week, 1–4 days/week, <1 day/week.

### 2.4. Statistical Analysis

As descriptive statistics, the baseline characteristics are summarized as mean (standard deviation (SD)) for continuous variables and frequency (percentage) for categorical variables for the total participants, men, and women. Changes in PA and social contacts over five years are shown as mean (95% confidence interval (CI)). Data of participants with missing values for PA or social contact were excluded from the descriptive statistics.

To evaluate the longitudinal associations of social contact with PA over five years, we used the parallel latent growth curve model, which is a multivariate statistical method within the structural equation modeling framework [19]. This approach allows estimating the latent growth parameters that indicate the baseline level of a variable (intercept) and the change in the variable over five years (slope), based on the observed social contact and PA in the three waves.

**Figure 2:**
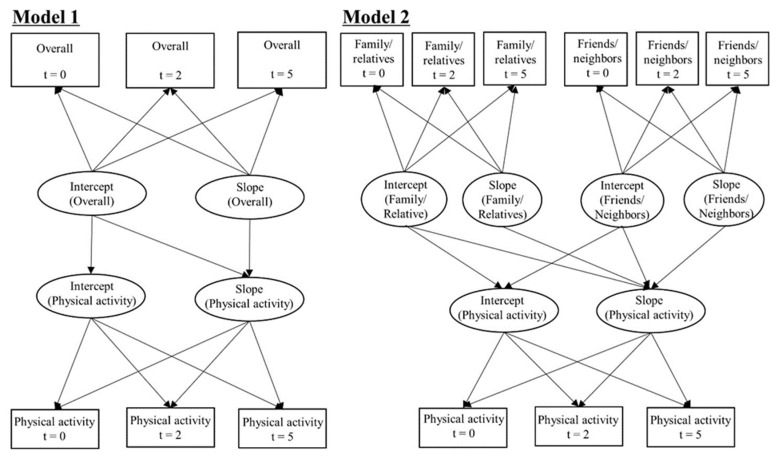
Parallel latent growth curve model fitted to the physical activity with social contact. Model 1 examined the association of frequency of overall social contact with physical activity over five years. Model 2 evaluated the association of each source of social contact with physical activity (family/relatives and friends/neighbors). The models were adjusted for sociodemographic variables (sex, age, years of education, subjective economic status, marital status, employment status), health status (self-rated health, mental health, medical history of diabetes and hypertension), and health behavior (smoking, alcohol, eating habits). Notes: In the figure, t represents the number of years elapsed since the baseline. Overall = frequency of contact with family/relatives/friends/neighbors; Family/Relatives = frequency of contact with family or relatives; Friends/Neighbors = frequency of contact with friends or neighbors.

We conducted two analysis models: Model 1 was fitted to PA with overall social contact; Model 2 was fitted to the PA with social contact sauce (family/relatives and friends/neighbors). Both models were adjusted for the covariates described above, and the unstandardized regression coefficient (β) and standard error (SE) were estimated. We examined the associations of social contact with PA, including the cross-sectional association (path from the intercept of social contact to the intercept of PA), prospective association (path from the intercept of social contact to the slope of PA), and parallel association (path from the slope of social contact to the slope of PA) in each model (Figure 2).

A previous study reported no sex differences in the relationships between social contact and PA [18]. Moreover, we performed multigroup analysis as the preliminary analysis and found no sex differences (Tables S1 and S2). Therefore, we highlight the results of parallel growth curve modeling, including all participants.

The model goodness-of-fit was evaluated using the comparative fit index (CFI), the Tucker–Lewis index (TLI), and the root mean square error of approximation (RMSEA). These indices indicate a good model fit to the data if CFI ≥ 0.90, TLI ≥ 0.90, and RMSEA ≤ 0.08 [32].

A full information maximum likelihood estimation was used for Models 1 and 2 of the latent growth curve models to account for missing data, assuming these were missing at random. *p*-values < 0.05 were considered statistically significant, and IBM SPSS AMOS version 25.0 was used for all statistical analyses (IBM Corp., Armonk, NY, USA).

## 3. Results

Participants’ baseline characteristics are shown in Table 1. The mean age (SD) of all participants was 51.1 (14.5) years, and 43.4% were men. The prevalence of missing values in each variable ranged from 0.1% to 4.7%. The prevalence of workers, regular drinkers, and smokers was much higher in men than in women, but the proportion of other variables showed equivalent (Table 1).

**Figure 3:**
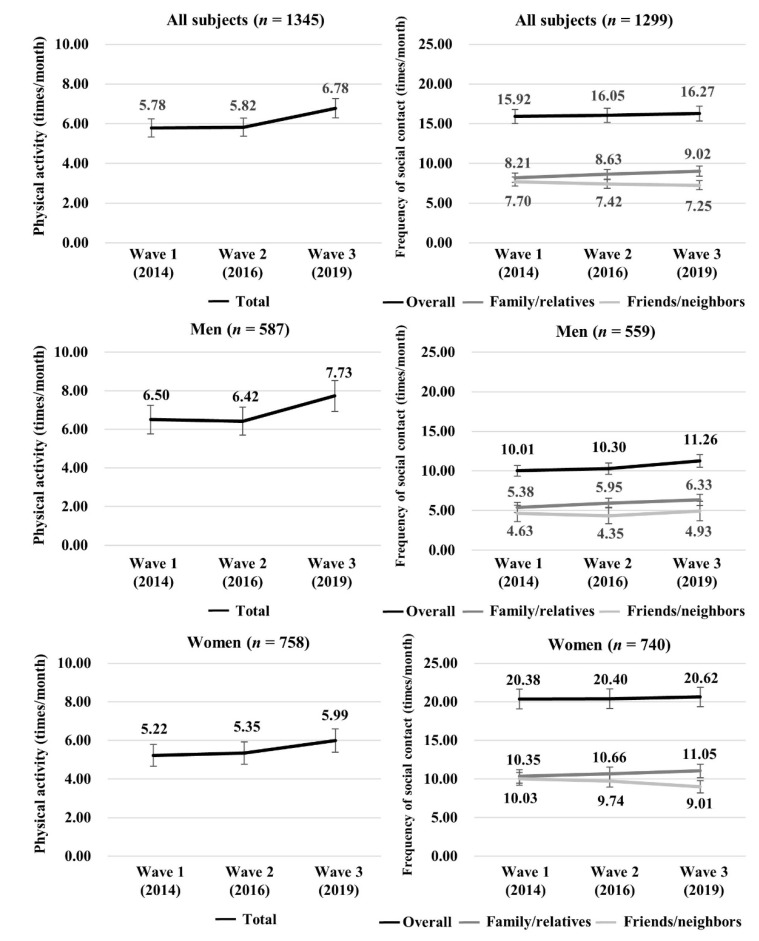
Changes in physical activity and social contact over five years among Japanese adults.

Changes in PA and social contact over five years are shown in Figure 3. The mean (95% CI) PA increased from 5.78 (5.32, 6.24) times to 6.78 (6.29, 7.27) times in all participants, from 6.50 (5.77, 7.24) times to 7.73 (6.94, 8.53) times in men, and from 5.22 (4.65, 5.80) times to 5.99 (5.39, 6.60) times in women. Regarding social contact, the overall frequency was slightly increased, from 15.92 (15.03, 16.81) times to 16.27 (15.35, 17.20) times in all participants; from 10.01 (8.98, 11.04) times to 11.26 (10.03, 12.48) times in men; and from 20.38 (19.11, 21.64) times to 20.62 (19.36, 21.89) times in women. The frequency of contact with family/relatives increased in each category, from 8.21 (7.62, 8.81) times to 9.02 (8.40, 9.64) times in all participants, from 5.38 (4.72, 6.05) times to 6.33 (5.53, 7.13) times in men, and from 10.35 (9.47, 11.23) times to 11.05 (10.17, 11.93) times in women. The frequency of contact with friends/neighbors decreased in all participants and women (all: from 7.70 (7.14, 8.27) times to 7.25 (6.69, 7.81) times, women: from 10.03 (9.20, 10.85) times to 9.01 (8.21, 9.82)). It remained nearly unchanged in men (4.63 (3.98, 5.28) times in wave 1 and 4.93 (4.22, 5.63) times in wave 3) (Figure 3).

The results of the latent growth curve model, examining the association of social contact with PA, are shown in Table 2. All fit indicators showed a good model fit to the data. Although there was no significant path from total social contact to the intercept of PA, we observed a significant path from the intercept (β (SE) = 0.009 (0.005)) and slope of overall social contact (β (SE) = 0.169 (0.071)) to the slope of PA (Table 2).

The results of the latent growth curve model, evaluating the associations of social contact sources with PA, are shown in Table 3. The model was also well fitted to the data. There was a significant path from the intercept of friends/neighbors to the intercept of PA (β (SE) = 0.104 (0.034)) and from the intercept and slope of family/relatives to the slope of PA (intercept: β (SE) = 0.016 (0.008), slope: β (SE) = 0.139 (0.065)) (Table 3).

## 4. Discussion

This study aimed to examine social contact and PA trajectories over five years and the longitudinal relationships. Our findings revealed that the associations of social contact with PA are differentially between the social contact sources; there was a positive cross-sectional association of contacting friends/neighbors with PA, and prospective and parallel positive associations of contacting family/relatives with PA.

### 4.1. Trajectories of PA and Social Contact over Five Years

Among the study population, PA increased over five years, which is inconsistent with previous studies [33]. This finding may reflect cross-cultural differences. According to a Japanese national survey [6], the older population is more likely to participate in exercise than the younger population. Moreover, subjective PA increases slightly with age among Japanese adults aged ≥60 [34]. Another possible reason is that discrete-time increases after retirement [22], and increased health awareness with age could increase PA [35].

The trajectories of social contact indicated differences between contact with family members and non-family members; the frequency of social contact with family/relatives was increased, whereas that with friends/neighbors was decreased. A previous study reported that the frequency of in-person contact with family remains relatively stable across the life course, but the frequency of visits with non-family members declines [23]. Although we measured social contact, including in-person and non-in-person contact, the previous study supports our findings. Life events, such as the birth of a child or caring for a parent, would increase contact with family members and decrease contact with friends.

### 4.2. Longitudinal Association of Social Contact with PA

We observed prospective and parallel associations between overall social contact and PA, consistent with a previous study [20]. Social contact could increase PA through multiple pathways. First, people with higher social contact have more varied sources of health-related information, which may help them adopt a healthy lifestyle [8]. Second, individuals with higher social contact may receive social support in exercising from their social network, which is a determinant of PA [13]. Third, social contact helps people maintain physical and mental health [16,36] and may increase PA levels. Several studies have reported that social contact can produce negative consequences, such as relational strain, when networks are characterized by a high degree of closure, lacking bridges to other networks [37]. However, a previous cross-sectional study suggested that adults with higher social strain are more likely to be physically active [38]. Individuals often use PA to cope with stressful situations, such as social strain [22]. In the current study, we could not detect underlying mechanisms in the observed associations; therefore, future research is needed to investigate this point.

Contact with family/relatives, and not with friends/neighbors, demonstrated a prospective and parallel association with PA. It may reflect differences in the direct and indirect effects of social contact on PA. Existing literature reports that the number of different types of social support from a family associated with PA is greater than the number from friends [13]. Family members share household roles and tasks, including taking care of children or parents, resulting in greater available time to PA. Other previous studies have suggested that frequent contact with family is related to better mental health, whereas there is no association between contact with friends and mental health among Japanese women [16]. Therefore, individuals with a higher degree of social contact with family/relatives at baseline and greater social contact with family/relatives are more likely to have increased PA over five years.

The cross-sectional association between contact with friends/neighbors and PA is consistent with a previous cross-sectional study [18,39]. However, there was no apparent prospective or parallel association between these behaviors. It may indicate reverse causality, with individuals who are physically active tending to contact friends/neighbors more frequently. Although a prior systematic review reported that peer-based intervention increases PA [40], increasing the frequency of contact with friends might not affect PA. The definition of peers, which share a common culture or health problems, is more restricted than that of friends/neighbors [40]. Therefore, increasing social contact among peers can produce social support for PA, whereas this may not be the case in facilitating social contact between friends/neighbors who are not peers. Our findings indicate that developing intervention programs that are focused on improving social interaction with family members could be effective for increasing long-term PA among community-dwelling adults.

### 4.3. Strengths and Limitations

The strengths of this study include its longitudinal design and observations made at three-time points, which enabled us to detect the trajectories of social contact and PA, and longitudinal relationships between social contact source and PA. A longitudinal design can identify the changes in PA owing to social contact sources and is more likely to suggest cause and effect relationships than a cross-sectional study, which is employed in most studies in this area. Additionally, we enrolled a community-representative population in the baseline survey; thus, this study would generalize our findings to other populations in the area with similar regional characteristics.

However, this study has several limitations that should be considered. First, since we conducted mail surveys at the three time points, sample attrition occurred. Although sample attrition is a feature of the longitudinal study, this might limit the generalizability of our findings. Second, because social contact and PA were self-reported, these behaviors might be overestimated. Third, we did not consider the quality of social networks. If social network members have a negative attitude toward PA, the influence from the network could be a barrier to PA [41]. Further studies should investigate the association of interaction between social contact and social network quality with PA.

## 5. Conclusions

Increasing PA level is essential for maintaining functional capacity and enhancing the quality of life. We identify social contact and PA trajectories over five years and the longitudinal relationships using a parallel latent growth curve model. The findings suggest that promoting social contact, especially contacting family/relatives, could increase PA. Although individuals with functional decline or a negative attitude toward PA are less likely to be physically active, they engage in PA through the influence of their social environment. Developing interventions to promote social contact with family/relatives would be vital to increasing the community-level PA.

## Figures and Tables

**Table 1:** Baseline characteristics of the analyzed sample.

Variables		All (*n* = 1395)		Men (*n* = 606)		Women (*n* = 789)	
Age—mean, SD		51.1	14.5	52.1	14.3	50.4	14.6
Age group—*n*, %							
	Young (20–39 years)	347	24.9	133	21.9	214	27.1
	Middle-aged (40–64 years)	777	55.7	347	57.3	430	54.5
	Older adults (≥65 years)	271	19.4	126	20.8	145	18.4
Years of education—*n*, %							
	<13 years	454	32.5	189	31.2	265	33.6
	≥13 years	875	62.7	385	63.5	490	62.1
	Missing	66	4.7	32	5.3	34	4.3
Subjective economic status—*n*, %							
	High	392	28.1	161	26.6	231	29.3
	Medium	545	39.1	232	38.3	313	39.7
	Low	429	30.8	202	33.3	227	28.8
	Missing	29	2.1	11	1.8	18	2.3
Marital status—*n*, %							
	Married	1087	77.9	481	79.4	606	76.8
	Divorced/widowed/single	278	19.9	113	18.6	165	20.9
	Missing	30	2.2	12	2.0	18	2.3
Employment status—*n*, %							
	Paid worker	994	71.3	503	83.0	491	62.2
	Not in the labor force	372	26.7	89	14.7	283	35.9
	Missing	29	2.1	14	2.3	15	1.9
Self-rated health—*n*, %							
	Good	1240	88.9	514	84.8	726	92.0
	Poor	148	10.6	89	14.7	59	7.5
	Missing	7	0.5	3	0.5	4	0.5
Mental health—mean, SD		8.2	2.9	7.8	2.9	8.5	2.9
Medical history—*n*, %							
	Diabetes mellitus	69	4.9	47	7.8	22	2.8
	Hypertension	212	15.2	111	18.3	101	12.8
Alcohol consumption—*n*, %							
	≥1 day/week	663	47.5	376	62.0	287	36.4
	<1 day/week	358	25.7	132	21.8	226	28.6
	Former-drinker	82	5.9	29	4.8	53	6.7
	Non-drinker	289	20.7	69	11.4	220	27.9
	Missing	3	0.2	0	0.0	3	0.4
Smoking status—*n*, %							
	Smoker	233	16.7	170	28.1	63	8.0
	Former-smoker	424	30.4	277	45.7	147	18.6
	Non-smoker	734	52.6	159	26.2	575	72.9
	Missing	4	0.3	0	0.0	4	0.5
Frequency of well-balanced meals—*n*, %							
	≥5 days/week	1030	73.8	427	70.5	603	76.4
	1–4 days/week	306	21.9	148	24.4	158	20.0
	<1 day/week	58	4.2	31	5.1	27	3.4
	Missing	1	0.1	0	0.0	1	0.1

SD, standard deviation.

**Table 2:** Estimated coefficients of the parallel latent growth curve model (Model 1) ^a^.

Parameter		All (*n* = 1395)		
		β	SE	*p*-Value
PA (Intercept)				
	Overall social contact (Intercept)	0.029	0.018	0.113
PA (Slope)				
	Overall social contact (Intercept)	0.009	0.005	0.044
	Overall social contact (Slope)	0.169	0.071	0.017
	CFI	0.995		
	TLI	0.989		
	RMSEA (90% CI)	0.014 (0.003, 0.021)		

β, unstandardized regression coefficient; SE, standard error; CFI, comparative fit index; TLI, Tucker–Lewis index; RMSEA, root mean square error of approximation; CI, confidence interval. ^a^ Parallel latent growth curve model included PA as the dependent variable, frequency of contact with family/relatives/friends/neighbors as independent variables, sociodemographic factors (age, sex, years of education, subjective economic status, marital status, employment status), health status (self-rated health, mental health, medical history), and health behavior (smoking status, alcohol consumption, eating habits) as covariates.

**Table 3:** Estimated coefficients of the parallel latent growth curve model (Model 2) ^a^.

Parameter		All (*n* = 1395)		
		β	SE	*p*-Value
PA (Intercept)				
	Frequency of contacting family/relatives (Intercept)	−0.022	0.025	0.386
	Frequency of contacting friends/neighbors (Intercept)	0.104	0.034	0.002
PA (Slope)				
	Frequency of contacting family/relatives (Intercept)	0.016	0.006	0.008
	Frequency of contacting friends/neighbors (Intercept)	0.002	0.008	0.783
	Frequency of contacting family/relatives (Slope)	0.139	0.065	0.033
	Frequency of contacting friends/neighbors (Slope)	0.154	0.113	0.174
	CFI	0.970		
	TLI	0.950		
	RMSEA (90% CI)	0.029 (0.025, 0.033)		

β, unstandardized regression coefficient; SE, standard error; CFI, comparative fit index; TLI, Tucker–Lewis index; RMSEA, root mean square error of approximation; CI, confidence interval. ^a^ Parallel latent growth curve model included PA as the dependent variable, frequency of contact with family/relatives and friends/neighbors as independent variables, sociodemographic factors (age, sex, years of education, subjective economic status, marital status, employment status), health status (self-rated health, mental health, medical history), and health behavior (smoking status, alcohol consumption, eating habits) as covariates.

## Data Availability

The data presented in this study are available on request from the corresponding author.

## Supplementary Materials

The following are available online at https://www.mdpi.com/1660-4601/18/5/2313/s1. Table S1: Estimated coefficients of the multigroup analysis (Model 1). Table S2: Estimated coefficients of the multigroup analysis (Model 2).
