# Supplementary material for: Social Contact with Family and Non-Family Members Differentially Affects Physical Activity: A Parallel Latent Growth Curve Modeling Approach
Source: Int J Environ Res Public Health. 2021 Feb 26;18(5):2313. doi: 10.3390/ijerph18052313 (PMC7956539; doi:10.3390/ijerph18052313)
Supplement: Supplementary file 1 [file ijerph-18-02313-s001.pdf]

Supplement table 1. Estimated coefficients of the multigroup analysis (Model 1)[a].

	Male (n=606)			Female (n=789)			Gender difference
	β	SE	P-value	β	SE	P-value	P-value
PA (Intercept)							
Overall social contact (Intercept)	0.030	0.040	0.445	0.087	0.022	<0.001	0.211
PA (slope)							
Overall social contact (Intercept)	0.015	0.010	0.136	-0.003	0.006	0.625	0.124
Overall social contact (Slope)	0.143	0.105	0.173	0.159	0.104	0.127	0.912
CFI	0.995						
TLI	0.986						
RMSEA (90% CI)	0.010 (0.001, 0.017)						

β, unstandardized regression coefficient; SE, standard error; CFI, comparative fit index; TLI, Tucker–Lewis index; RMSEA, root mean square error of approximation; CI, confidence interval.

[a] Parallel latent growth curve model included physical activity as the dependent variable, frequency of contact with family/relatives/friends/neighbors as independent variables, sociodemographic factors (age, years of education, subjective economic status, marital status, employment status), health status (self-rated health, mental health, medical history), and health behavior (smoking status, alcohol consumption, eating habits) as covariates.

Supplement table 2. Estimated coefficients of the multigroup analysis (Model 2) [a].

	Male (n=606)			Female (n=789)			Gender difference
	β	SE	P-value	β	SE	P-value	P-value
PA (Intercept)							
Frequency of contact with family/relatives (Intercept)	-0.078	0.056	0.164	0.031	0.030	0.307	0.087
Frequency of contact with friends/neighbors (Intercept)	0.209	0.084	0.013	0.155	0.019	<0.001	0.624
PA (Slope)							
Frequency of contact with family/relatives (Intercept)	0.023	0.014	0.100	0.003	0.007	0.644	0.215
Frequency of contact with friends/neighbors (Intercept)	0.010	0.022	0.656	-0.010	0.009	0.283	0.412
Frequency of contact with family/relatives (Slope)	0.574	0.628	0.360	0.223	0.154	0.147	0.582
Frequency of contact with friends/neighbors (Slope)	0.024	0.116	0.833	0.110	0.161	0.495	0.667
CFI	0.969						
TLI	0.937						
RMSEA (90% CI)	0.022 (0.018, 0.026)						

β, unstandardized regression coefficient; SE, standard error; CFI, comparative fit index; TLI, Tucker–Lewis index; RMSEA, root mean square error of approximation; CI, confidence interval.

[a] Parallel latent growth curve model included physical activity as the dependent variable, frequency of contact with family/relatives and friends/neighbors as independent variables, sociodemographic factors (age, years of education, subjective economic status, marital status, employment status), health status (self-rated health, mental health, medical history), and health behavior (smoking status, alcohol consumption, eating habits) as covariates.
